# Microbial dysbiosis and childhood asthma development: Integrated role of the airway and gut microbiome, environmental exposures, and host metabolic and immune response

**DOI:** 10.3389/fimmu.2022.1028209

**Published:** 2022-09-30

**Authors:** Conglin Liu, Heidi Makrinioti, Sejal Saglani, Michael Bowman, Lih-Ling Lin, Carlos A. Camargo, Kohei Hasegawa, Zhaozhong Zhu

**Affiliations:** ^1^ Immunology & Inflammation Research Therapeutic Area, Sanofi US, Cambridge, MA, United States; ^2^ London School of Paediatrics, London, United Kingdom; ^3^ National Heart and Lung Institute, Imperial College, London, United Kingdom; ^4^ Centre for Paediatrics and Child Health, Imperial College, London, United Kingdom; ^5^ Department of Emergency Medicine, Massachusetts General Hospital, Harvard Medical School, Boston, MA, United States

**Keywords:** microbial dysbiosis, airway microbiome, gut microbiome, immune mechanism, metabolic mechanism, childhood asthma

## Abstract

Asthma is a chronic and heterogeneous respiratory disease with many risk factors that typically originate during early childhood. A complex interplay between environmental factors and genetic predisposition is considered to shape the lung and gut microbiome in early life. The growing literature has identified that changes in the relative abundance of microbes (microbial dysbiosis) and reduced microbial diversity, as triggers of the airway-gut axis crosstalk dysregulation, are associated with asthma development. There are several mechanisms underlying microbial dysbiosis to childhood asthma development pathways. For example, a bacterial infection in the airway of infants can lead to the activation and/or dysregulation of inflammatory pathways that contribute to bronchoconstriction and bronchial hyperresponsiveness. In addition, gut microbial dysbiosis in infancy can affect immune development and differentiation, resulting in a suboptimal balance between innate and adaptive immunity. This evolving dysregulation of secretion of pro-inflammatory mediators has been associated with persistent airway inflammation and subsequent asthma development. In this review, we examine current evidence around associations between the airway and gut microbial dysbiosis with childhood asthma development. More specifically, this review focuses on discussing the integrated roles of environmental exposures, host metabolic and immune responses, airway and gut microbial dysbiosis in driving childhood asthma development.

## Introduction

Asthma is one of the most common chronic respiratory diseases affecting more than 300 million individuals worldwide (1). The prevalence of asthma remains high, mainly due to high incidence in the first years of life (2, 3). Asthma is characterized by symptoms of wheeze, shortness of breath, chest tightness, cough, and expiratory airway limitation (1). Epidemiological studies have suggested many risk factors for asthma development, but mainly representing three domains: demographics (e.g., age, sex, and family history), genetics, and environmental exposures (e.g., bacterial and viral infection, air pollution, and diet) (3–6). The interplay between these risk factors underlies the pathobiological mechanisms of asthma and contributes to the variability in pathogenic mechanisms and in response to treatment. It is accepted that asthma consists of a range of subtypes differing in presentation and pathobiological mechanisms (7), such as allergic vs. non-allergic asthma (8–10) and childhood- vs. adult-onset asthma (9, 11–13). As expected, the effects of risk factors on asthma incidence vary between asthma subtypes. Thus, it is critical to identify asthma subtype-specific risk factors. Of the various risk factors for childhood asthma development, microbial dysbiosis in the airway and gut may play a crucial role given their complex interplay with host genetic susceptibility, environmental exposures, and metabolic and immune response (14–16). For several reasons, such complex interplay becomes the most important for asthma during early-life development. First, early life is a crucial period in which the gene-environmental interaction helps to shape immune development (17). Second, most infections occur in infancy, and often are recurrent episodes and are relatively severe, which have long-term respiratory sequela in later life (18). Third, early life is a critical period of airway development, which is crucial in determining lung function and respiratory diseases in later childhood and adulthood (19). This review will examine the current epidemiological evidence about the relationship between airway and gut microbial dysbiosis and childhood asthma development, their integrated roles with environmental exposures, and the metabolic and immune mechanisms underlying airway and gut microbiota diversity and asthma development in childhood.

## Human airway and gut microbiota

The human microbiota contains 10-100 trillion microbes harbored by each person (20). The microbes are distributed across several major body sites, including oral cavity, respiratory system, gastrointestinal system, vagina, and skin (21), with the microbial composition varying depending on the body site. Such distinct microbial composition is determined based on genetic susceptibility and environmental exposures (22). The interplay of these factors underlies the pathobiological mechanisms for diseases, such as asthma (16). Growing evidence suggests that microbiota in the respiratory system consists of four major pathogenic bacterial phyla, including Actinobacteria, Bacteroidetes, Firmicutes, and Proteobacteria (21). The gastrointestinal system’s microbiota consists of six major bacterial phyla, including Actinobacteria, Bacteroidetes, Firmicutes, Fusobacteria, Proteobacteria, and Verrucomicrobia (21). The cross-talk between airway and gut microbiota can have synergistic effects on the development of respiratory diseases. Disturbances in gut microbial composition limit the capacity to modulate adequate immune responses, which not only has been linked to inflammatory conditions in the gastrointestinal tract itself but also in the airway, referred to as the “gut-lung axis” (23–25). The importance of such interaction has become more evident with the identification of microbe-produced metabolites in both the airway and gut. These metabolites can contribute to various respiratory diseases, such as asthma (26–29). Yet, until recently, the mechanisms that underlie the link between microbial dysbiosis in the airway and gut and childhood asthma development remained unclear.

## Airway microbiota and childhood asthma development

Growing evidence suggests that the airway microbiota in early life is associated with childhood asthma development (29–41). For example, an early study from the Copenhagen Prospective Study on Asthma in Childhood (COPSAC)—a prospective birth cohort—has reported that pathogenic bacterial colonization (e.g., *Haemophilus influenzae*, *Moraxella catarrhalis*, and *Streptococcus pneumoniae*) of the nasopharynx in infants is associated with asthma development by age 5 years (30). Likewise, a study from Childhood Asthma Study (CAS)—a prospective birth cohort from Australia—has also found that *Streptococcus* colonization in infants’ upper airway is strongly associated with asthma development by age 5 years (32). In addition, a recent study from the 35th Multicenter Airway Research Collaboration (MARC-35)—a prospective cohort of infants with severe bronchiolitis—has used a dual-transcriptome (metatranscriptome and transcriptome) approach and found that a higher abundance of *Streptococcus pneumoniae* in the nasopharyngeal airway is associated with a greater risk of developing asthma by age 6 years, particularly in infants with non-rhinovirus infection (29). And the major bacterial species drive the asthma-related microbial functional pathways, such as fatty acid metabolism and glycolysis pathways (29), which have been found to play important roles in asthma pathobiology (9, 42–44). The abundance of major bacterial species is also associated with asthma-related host transcripts (e.g., *DAGLB*) (29). Furthermore, a recent study from the Steps to the Healthy Development and Well-being of Children (STEPS)—a prospective population-based birth cohort study—has reported that infants with persistent *Moraxella* sparsity have a higher risk of asthma development by age 7 years (34). Finally, a study from COPSAC 2010 (COPSAC_2010_) has reported that an increased α-diversity (i.e., within-sample measures of similarity or dissimilarity) and higher abundance of *Veillonella* and *Prevotella* in the airway at age 1 month are associated with asthma development by age 6 years (33). These studies collectively suggest the important roles of airway microbiota from early life in the development of childhood asthma.

## Gut microbiota and childhood asthma development

Compared with the airway microbiota, it seems less intuitive that the gut microbiota would be connected with respiratory diseases since gut and lungs differ anatomically. Yet, alterations in gut microbial composition may have a notable effect on respiratory diseases, such as asthma, by shaping microbial communities and modulating the metabolic and immune response, a so-called “gut-lung axis” concept (23–25). Growing evidence suggests that gut microbiota in early life is associated with childhood asthma development (26–28, 36, 45–50). For example, a study from COPSAC_2010_ has found that immature gut microbial composition (measured by microbiota age) in children at age 1 year is associated with a higher risk of asthma development by age 5 years (47). Such association is stronger in children who have mothers with asthma, indicating the synergistic effect between genetic predisposition and inadequate gut microbial stimulation on asthma development (47). Additionally, a study from the Wayne County Health, Environment, Allergy and Asthma Longitudinal Study (WHEALS)—a prospective birth cohort—has found that lower relative abundance of beneficial bacteria in neonatal gut (e.g., *Akkermansia*, *Bifidobacterium*, and *Faecalibacterium*) are associated with a higher risk of asthma by age 4 years (26). Furthermore, a recent study from the Vitamin D Antenatal Asthma Reduction Trial (VDAART)—a randomized trial on the effects of prenatal vitamin D supplementation on asthma in offspring—has found that a higher level of *Veillonella* and histidine pathway metabolites or a lower level of *Oscillospiraceae* UCG-005 in gut of children at age 3 years are associated with an increased wheeze frequency between ages 3 and 5 years (28). In contrast, a mature gut microbial composition in early life may help to lower the risk of childhood asthma development. For example, a recent study from the Protection against Allergy-Study in Rural Environments (PASTURE)—a prospective birth cohort—has found that a mature gut microbial composition in infants from age 2 to 12 months—consisting of *Bacteroides, Coprococcus*, *Roseburia*, and *Turicibacter*—can produce short-chain fatty acids (SCFAs), such as butyrate, which have a protective effect on asthma development by 6 years (48). Likewise, a recent study from the Canadian Healthy Infant Longitudinal Development (CHILD)—a prospective birth cohort—has found that an increased α-diversity in gut microbiota due to decreased antibiotic use in infancy is associated with a reduced risk of asthma development by age 5 years (49). Taken together, these diverse pieces of evidence provide support for the relation of gut microbiota in early life and development of childhood asthma.

## Integrated roles of environmental exposures and microbiota on childhood asthma development

**Figure 1:**
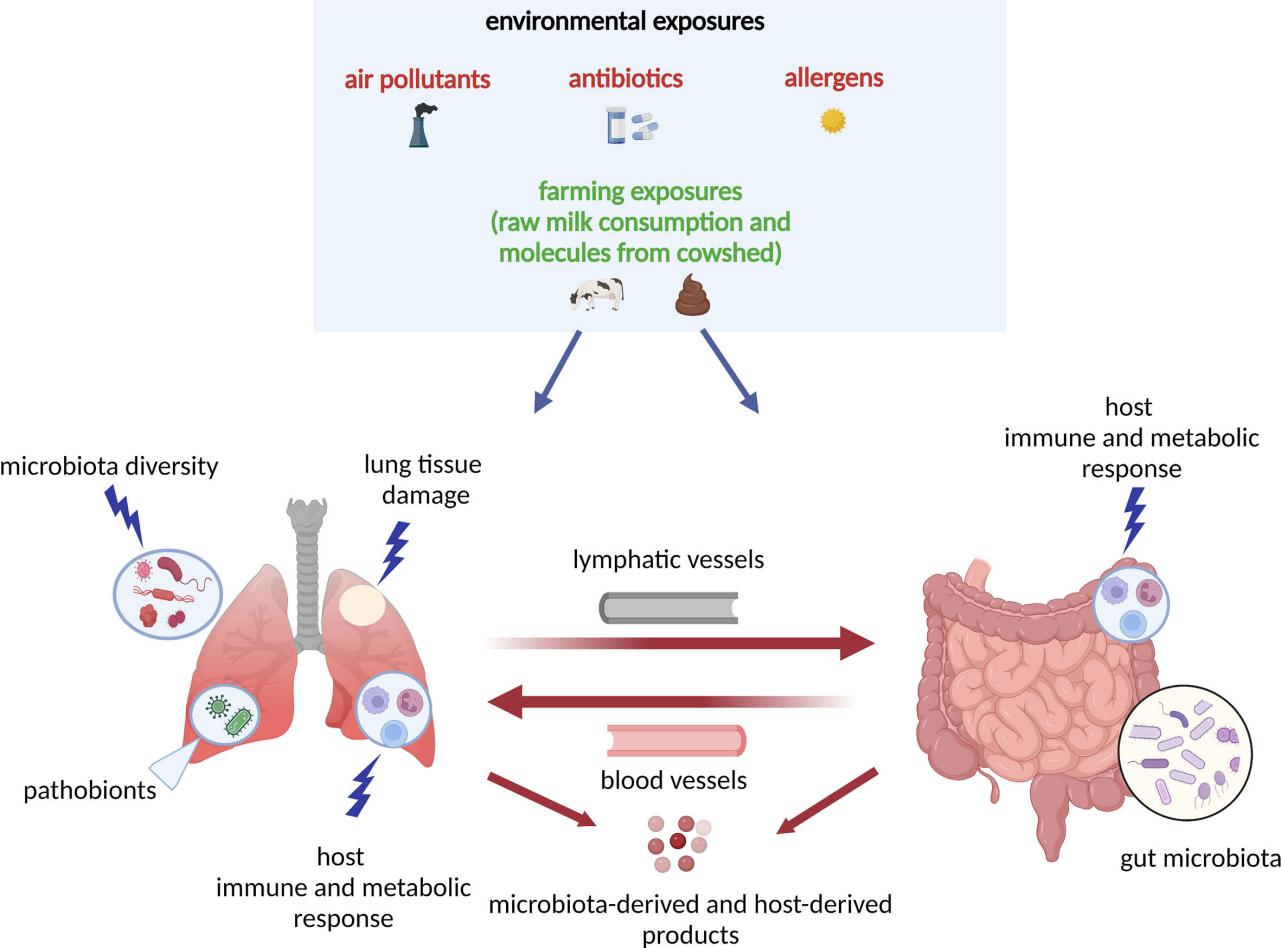
Model of the host–microbiota interaction during dysbiosis as a pathogenetic mechanism underlying asthma development: the “gut-lung axis”. A variety of environmental exposures, either protective (green color) or non-protective (red color) can trigger microbial dysbiosis (i.e., the alteration of the function and composition of the microbiota). In this model, in response to the non-protective environmental exposures (air pollutants, antibiotics, allergens), there is lung tissue damage and release of molecules that interact with host immune and metabolic pathways. The lung and gut microbiota diversity is perturbed, and there is increase in pathobionts (i.e., potentially disease-causing organisms that under normal circumstances act as symbionts) in both lung and gut tissues. In response to microbial dysbiosis, there is also increased activation of leukocytes and other innate-mediated soluble factors, which circulate through blood and lymphatic vessels and trigger adaptive immune responses with T_H_1, T_H_2, T_H_17, Treg differentiation. All host- and microbiota‐derived (e.g., SCFAs, cytokines and chemokines) products act at the local (lung) or distal (gut) levels *via* circulation through blood and lymphatic vessels. The “gut-lung axis” refers to bidirectional crosstalk between these two mucosal sites of the body as described above. SCFAs, short-chain fatty acids; T_H_2, T helper 2; T_H_17, T helper 17; Treg, regulatory T cell.

The complex interplay between environmental exposures and microbiota underlies the pathobiological mechanisms for asthma, especially in early life (**Figure 1**). One prominent example of environmental exposure affecting asthma development is the farm effect. Many epidemiological studies have found living on a farm in early life is associated with reduced risk of childhood asthma development (48, 51–54). For example, an early study has investigated two farm populations (Amish and Hutterites) with similar genetic backgrounds but different farming practices and found a distinct risk for childhood asthma development (52). The Amish follow traditional farming practices (i.e., high microbial exposures to animals) whereas the Hutterites use industrialized farming practices. The study has found the environment from Amish farms protects children against asthma development (52). On the other hand, a recent study from COPSAC_2010_ found that an airway and gut microbial signature due to urbanization (i.e., no farm effect) in infancy increases the risk of asthma, eczema, and allergic sensitization at age 6 years (55). Collectively, these interesting results suggest that early-life microbial farm animal exposures may lower the risk of childhood asthma development, perhaps by shaping the innate immune response.

In addition to farm environmental exposures, other environmental exposures (e.g., antibiotics use, diet) may also have an effect on childhood asthma development by modulating the airway and gut microbiota in children’s early life. For example, a recent study from the STEPS study has found that exposures to antibiotics within the first year of life are associated with increased risk of asthma development by age 7 years; and such effect is partially mediated by longitudinal changes in the nasal airway microbiota characterized as a low asthma risk profile with persistent *Moraxella* dominance vs a high asthma risk profile with early *Moraxella* sparsity (56). Also, a study from the Urban Environment and Childhood Asthma (URECA)—a longitudinal birth cohort—has reported that the exposure to both allergens and the bacterial species (primarily from Bacteroidetes and Firmicutes phyla) within the first year of life can protect from atopy and recurrent wheeze development by age 3 years (57). Additionally, a study from the Infant Susceptibility to Pulmonary Infections and Asthma Following Respiratory Syncytial Virus Exposure (INSPIRE)—a longitudinal observational birth cohort—has found that breastfeeding during infancy has several beneficial effects on childhood asthma development by reducing the dose-response effect of the feeding on the ⍺-diversity of the early-life upper airway and gut microbiota, and protects children from the development of lower respiratory tract infections in infancy, and asthma at age 4 years (41). Moreover, a recent study from COPSAC_2010_ has found that if the gut microbial signature at age 1 year is retained from a cesarean section delivery period, there is a higher risk of asthma development by age 6 years, but not at a higher risk if the gut microbial signature became mature (e.g., a higher abundance of *Akkermansia*, *Bacteroides*, and *Ruminococcus*) (50). This finding indicates the maturation of gut microbial composition may mitigate the effect of cesarean section delivery on childhood asthma development. These studies collectively suggest that it is crucial to consider the integrated roles of environmental exposures and microbiota from early life in the childhood asthma development.

## Immune mechanisms for airway microbiota and asthma link

**Figure 2:**
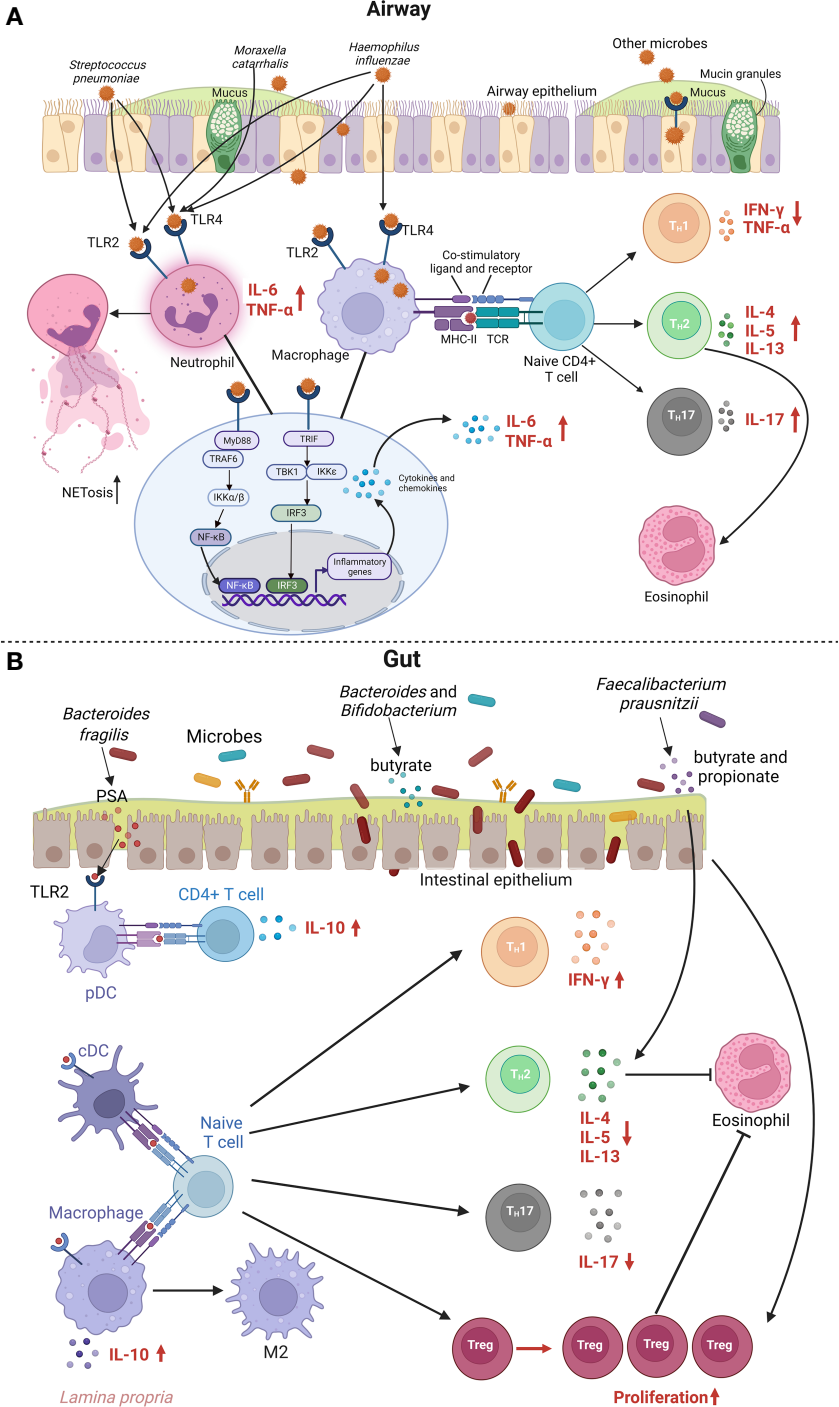
Metabolic and immune mechanisms for the link between airway or gut microbiota and asthma. **(A)** shows immune mechanisms for the airway epithelial cell signaling in response to bacterial pathogens. Various bacterial pathogens bind to innate sensors TLR2 and TLR4 and further activate MyD88- and TRIF-dependent pathways. MyD88 recruits TRAF6, which further activates the IKK complex, allows NF-кB to translocate into the nucleus, and leads to the overall production of inflammatory cytokines and chemokines, and activation of T cells. Additionally, recognition of various bacterial pathogens activates the TRIF-dependent signaling pathway, which involves the recruitment of TRIF, that leads to subsequent activation of TBK1 and IKKϵ along with induction of transcription factor IRF3. This signaling pathway results in interferon-related cytokines, and can potentiate NF-κB gene transcription. Enhanced neutrophil TLR2 and TLR4 signaling by bacterial pathogens promote neutrophils cytokine production and NETosis, a program for formation of NETs. Bacterial pathogens can also be recognized by TLR2 and TLR4 on macrophages, leading to activation of NF-κB and IRF3 signaling pathway and secretion of inflammatory mediators. Macrophages can also function as APC and regulate T cell activation. The T cell is presented an antigen with MHC II by APC. The recognition of the antigen-MHC II complex and the co-stimulatory molecules activates the T cell and leads downstream to differentiation into T_H_2 and T_H_17 cells, that can release various cytokines such as IL-4, IL-5 and IL-13, which lead to eosinophilic inflammation. APC, antigen-presenting cell; IKK, inhibitory kappa B kinases; IL, interleukin; IRF3, interferon regulatory factor 3; MHC, major histocompatibility complex; MyD88, myeloid differentiation primary response protein 88; NETosis, neutrophil extracellular trap formation; NETs, neutrophil extracellular traps; NF-кB, nuclear factor kappa B; TBK1, TANK-binding kinase 1; T_H_2, T helper 2; T_H_17, T helper 17; TLR, Toll-like receptor; TRAF6, tumor necrosis factor receptor associated factor 6; TRIF, TIR-domain-containing adapter-inducing interferon-β. **(B)** shows metabolic and immune mechanisms for the link between gut microbiota and asthma. PSA from *Bacteroides fragilis* induces and ligates TLR2 on pDC, which stimulate anti-inflammatory cytokine IL-10 secretion by CD4+ T cells. cDC and macrophage bound by gut microbiota show impaired ability to promote TH2- and TH17-type responses and tend to promote TH1-type responses and Treg proliferation, which lead to decreased eosinophilic inflammation. IL-10 dependent reprogramming of tissue macrophages is also essential for resolving inflammation by promoting M2 macrophage polarization. *Bacteroides* and *Bifidobacterium* can digest the fiber and produce SCFAs, such as butyrate. *Faecalibacterium prausnitzii* increases SCFAs level, such as butyrate and propionate, which leads to reduced levels of IL-4, IL-5 and IL-13, and elevated level of Tregs. cDC, classical dendritic cell; IFN, interferon; IL, interleukin; pDC, plasmacytoid dendritic cell; PSA, polysaccharide A; SCFAs, short-chain fatty acids; T_H_2, T helper 2; T_H_17, T helper 17; TLR, Toll-like receptor; Treg, regulatory T cell.

Microorganisms can present in host (e.g., airway) without interaction with the host, i.e., colocalization. Once there is an imbalance in the disease-related microbial community and interaction with host (i.e., microbial dysbiosis), host immune system can respond to the pathogenic microbes and lead to the local or/and systematic inflammation (14). Underlying mechanisms of the airway microbial dysbiosis and asthma link warrant further clarification. **Figure 2A** illustrates the major host immune mechanisms for airway epithelial cell signaling in response to bacterial pathogens. First, many studies have reported that innate immune responses play direct roles in host defense during the early stages of a respiratory infection, and they also exert a profound influence on the generation of the adaptive immune responses that ensue and on driving long-term respiratory sequela (most commonly asthma) (58, 59). The airway epithelium is a physical barrier and the first point of contact for inhaled pathogens (60). Notably, the airway epithelium contains a wide variety of pattern recognition receptors and antimicrobial compounds (e.g., mucins) that establish innate immunity (60). The pattern recognition receptors in the airway epithelium, such as Toll-like receptors (TLRs), are activated by invading pathogens, bacterial virulence factors, and endogenous mediators released due to airway tissue damage (61). The innate immune recognition of *Haemophilus influenzae*, *Moraxella catarrhalis*, and *Streptococcus pneumoniae*, mainly involves TLR2 and TLR4. These TLRs activate the transcription factor nuclear factor kappa B (NF-кB) and interferon regulatory factor 3 (IRF3) (61), which regulates the expression of inflammatory genes and the release of cytokines and chemokines related to asthma (62, 63), such as interleukin (IL)-6 and tumor necrosis factor (TNF)-α. For example, an *in vivo* study using an ovalbumin (OVA)-induced mouse model of allergic asthma has demonstrated that dysfunction of TLR4-positive innate immune cells including neutrophils and macrophages during *Haemophilus influenzae* infection promotes bacterial persistence and leads to the development of steroid-resistant neutrophilic asthma (64). In addition, an *in vitro* study has also shown that chronic colocalization with *Haemophilus influenzae* can induce neutrophil extracellular trap formation (NETosis) and releases soluble IL-6 receptor (sIL-6R) and IL-6 from neutrophils, which also associated with higher lung epithelium expression of TLR2 and TLR4 (65). The causal relationship between sIL-6R and childhood asthma is also supported by a recent Mendelian randomization study (66).

Second, in addition to the innate immunity, growing evidence suggests that adaptive immunity plays a pivotal role in the underlying mechanisms of the airway microbiota and asthma link (14, 67). For example, a recent *in vivo* study using a house dust mite (HDM)-challenged mouse model of the allergic airway inflammation has found that airway infection of *Streptococcus pneumoniae* leads to increased number of activated T helper 2 (T_H_2) cells and elevated level of T_H_2 cytokines, such as IL-4, IL-5 and IL-13 (68). Additionally, an *in vivo* study using HDM-challenged mouse model has found that airway infection with *Moraxella catarrhalis* triggers a strong inflammatory response with neutrophilic infiltrates, such as high amounts of IL-6 and TNF-α and moderate levels of CD4+ T-cell-derived interferon (IFN)-γ and IL-17. If bacterial infection occurs during HDM allergen sensitization, the allergic airway response is exacerbated, particularly by the expansion of T helper 17 (T_H_17) cells and increased TNF-α levels (69). In addition, a study using human bronchoalveolar lavage samples has demonstrated that a high bacterial load and supraglottic predominant taxa (e.g., *Prevotella* and *Veillonella*) is associated with an increased number of CD4+ IL-17+ T cells, and cytokines (IL-1β and IL-6) or chemokine (fractalkine) related to T_H_17 differentiation (70). Finally, an *in vivo* study using a HDM-challenged neonatal mouse model has demostrated that a dynamic change in lung microbiota in the first 2 weeks of life, from a dominance of Gammaproteobacteria and Firmicutes towards Bacteroidetes, is associated with an increased level of regulatory T cells (Tregs) and reduced aeroallergen responsiveness (71). Such findings indicate that the maturation of airway microbiota in early life is crucial to reduce the risk of developing allergic airway inflammation in later life. Notwithstanding the complexity of these mechanisms, the identification of the airway microbiome-host immune response interaction and its contribution to asthma pathobiology will likely prove important to future efforts to prevent childhood asthma.

## Metabolic and immune mechanisms for gut microbiota and asthma link

The mechanisms underlying a link between gut microbiota and asthma involve complex interactions between microbes, metabolites, and host immune responses (14). **Figure 2B** illustrates the major metabolic and immune mechanisms for the link between gut commensal bacteria and asthma. In the gut, commensal bacteria help to shape the cellular and physical maturation of both innate and adaptive immunity in early life and have profound effects on asthma pathobiology (72, 73). More specifically, the presence of specific microbial species helps maintain the gut barrier function by preserving tight junction formation at the gut epithelium, and by modulating immune responses to allergens (74). For example, an early *in vivo* study of a mouse model showed polysaccharide A from *Bacteroides fragilis* induces and ligates TLR2 on plasmacytoid dendritic cells, which is priming IL-10 producing T cells with potential anti-inflammatory properties (75). Additionally, another *in vivo* study using an HDM-challenged mouse model has found that high-fiber diet increases the abundance of *Bacteroides* and *Bifidobacterium*, which can digest the fiber and produce SCFAs, such as butyrate (76). Butyrate can decrease excessive inflammation through downregulating the secretion of pro-inflammatory mediators (e.g., IL-6, TNF-a) and activating IL-10 producing T cells and macrophages (77). Consequently, the study has found that mice fed a high-fiber diet have increased concentration of circulating SCFAs and decreased airway hyperresponsiveness (AHR) and allergic airway inflammation (76). A recent study has also demonstrated that infants who are breastfed and given *Bifidobacterium infantis* EVC001, has reduced intestinal T_H_2 and T_H_17 cytokines and increased IFN-β level (78). Furthermore, HDM-challenged mice given with *Faecalibacterium prausnitzii* have increased SCFAs (e.g., butyrate, propionate) level, which leads to reduced levels of IL-4, IL-5 and IL-13, and elevated level of Tregs, consequently alleviating the symptoms of allergic asthma (79). Finally, intervention studies have provided mechanistic evidence on gut commensal bacteria and immunoregulation. For example, a recent *in vivo* study of OVA-induced murine allergic airways disease has showed that probiotic administration (e.g., *Akkermansia*) alleviate the airway inflammation in mice with a genetic predisposition for airway inflammation (80). The study has found that probiotic bacteria—such as *Akkermansia*—can produce acetate, another SCFA that can inhibit NF-кB activity (80). The inhibition of NF-кB is known to alleviate airway inflammation in asthma by reducing T_H_2 cytokine production, such as IL-5 and IL-13 (81). The biodiversity intervention (i.e., more nature-oriented environment) may modify the gut microbiota in children (e.g., *Faecalibacterium*), which was associated with changes in plasma cytokine and Tregs levels (82). This finding suggests the biodiversity intervention improved immunoregulatory pathways in children and can potentially lower the risk of immune-mediated diseases (e.g., asthma) in urban societies (82).

## Conclusion and future directions

In this review, we have examined a broad range of major epidemiological and mechanistic studies and summarized the current evidence for the link between airway or gut microbiota and childhood asthma development. We also discussed some of the metabolic and immunological mechanisms underlying the link between microbiome exposure in early life and childhood asthma development. Nearly all microbiome-asthma studies have investigated either airway or gut microbiota – but not both. Thus, the synergistic effect of the airway and gut microbiota on childhood asthma development remains largely unclear (36, 83). We suggest future research shall examine the integrated effect of airway and gut microbiota on childhood asthma development in the same individual. Also, majority of mechanistic studies are in murine models, and often not early life models. The next step is to consider intervention studies with mechanisms built in. Additionally, we believe that integrating microbiome with other omics data—such as genomics, epigenomics, transcriptomics, proteomics, and metabolomics—will provide further insights into the pathobiology of asthma and its subtypes. Taken together, these efforts will facilitate the development of an early life microbiome-targeted prevention and intervention strategies for the primary prevention of childhood asthma (84).

## Author contributions

CL conducted the literature review, drafted the initial manuscript, designed the figure, and approved the final manuscript as submitted. HM designed the figure, reviewed the initial manuscript, and approved the final manuscript as submitted. SS, MB, and L-LL reviewed the initial manuscript and approved the final manuscript as submitted. CC and KH conceptualized the study, reviewed the initial manuscript, and approved the final manuscript as submitted. ZZ conceptualized and supervised the study, obtained funding, conducted the literature review, drafted the initial manuscript, designed the figure, and approved the final manuscript as submitted.

## Funding

This study was supported by the following grants: the National Institutes of Health: K01 AI-153558 (ZZ); Massachusetts General Hospital Department of Emergency Medicine Fellowship/Eleanor and Miles Shore Faculty Development Awards Program (ZZ); and the Harvard University William F. Milton Fund (ZZ).

## Conflict of interest

CL, MB, and L-LL are employees of Sanofi US and may hold shares and/or stock options in the company. CC and KH report grants from National Institutes of Health outside the submitted work. ZZ reports grants from National Institutes of Health and Harvard University during the conduct of the study.

The remaining authors declare that the research was conducted in the absence of any commercial or financial relationships that could be construed as a potential conflict of interest.

## Publisher’s note

All claims expressed in this article are solely those of the authors and do not necessarily represent those of their affiliated organizations, or those of the publisher, the editors and the reviewers. Any product that may be evaluated in this article, or claim that may be made by its manufacturer, is not guaranteed or endorsed by the publisher.
